# A comprehensive analysis of the chorion locus in silkmoth

**DOI:** 10.1038/srep16424

**Published:** 2015-11-10

**Authors:** Zhiwei Chen, Junko Nohata, Huizhen Guo, Shenglong Li, Jianqiu Liu, Youbing Guo, Kimiko Yamamoto, Keiko Kadono-Okuda, Chun Liu, Kallare P. Arunkumar, Javaregowda Nagaraju, Yan Zhang, Shiping Liu, Vassiliki Labropoulou, Luc Swevers, Panagiota Tsitoura, Kostas Iatrou, Karumathil P. Gopinathan, Marian R. Goldsmith, Qingyou Xia, Kazuei Mita

**Affiliations:** 1 State Key Laboratory of Silkworm Genome Biology, Chongqing 400716, China; 2 Kirin Brewery Co. Ltd, Toride, Japan; 3 National Institute of Agrobiological Sciences, Tsukuba 305-8634, Japan; 4 Centre for DNA Fingerprinting and Diagnostics, Hyderabad 500001, India; 5 Insect Molecular Genetics and Biotechnology, Institute of Biosciences & Applications, National Centre for Scientific Research “Demokritos”, Athens 15310, Greece; 6 Indian Institute of Science, Bangalore, India; 7 University of Rhode Island, Kingston 02881, USA

## Abstract

Despite more than 40 years of intense study, essential features of the silkmoth chorion (eggshell) are still not fully understood. To determine the precise structure of the chorion locus, we performed extensive EST analysis, constructed a bacterial artificial chromosome (BAC) contig, and obtained a continuous genomic sequence of 871,711 base pairs. We annotated 127 chorion genes in two segments interrupted by a 164 kb region with 5 non-chorion genes, orthologs of which were on chorion bearing scaffolds in 4 ditrysian families. Detailed transcriptome analysis revealed expression throughout choriogenesis of most chorion genes originally categorized as “middle”, and evidence for diverse regulatory mechanisms including cis-elements, alternative splicing and promoter utilization, and antisense RNA. Phylogenetic analysis revealed multigene family associations and faster evolution of early chorion genes and transcriptionally active pseudogenes. Proteomics analysis identified 99 chorion proteins in the eggshell and micropyle localization of 1 early and 6 Hc chorion proteins.

As a complex extracellular structure, the eggshell of *Bombyx mori* plays a significant role in the development of the oocyte and embryo. First, it sustains the structure of the oocyte and provides a passageway for sperm to enter the egg for fertilization. Secondly, it protects the developing embryo from external environmental hazards after oviposition and prevents it from drying out, while facilitating respiration.

The eggshell is mainly comprised of chorion proteins, which are synthesized and secreted only by follicular cells located in a series of 8 pupal ovarioles in *B. mori*^1^ (Supplementary Fig. 1A). The follicle itself consists of a single layer of polyploid epithelial cells, the follicular epithelium, which surrounds the oocyte (Supplementary Fig. 1B), and a separate cap of nurse cells that degenerates prior to choriogenesis, when most follicular cell biosynthetic activity is devoted to chorion protein production^2^. Chorion proteins are encoded by a multigene family that expresses more than 100 low-molecular weight structural proteins in a characteristic sequence during the early, middle, and late stages of choriogenesis^3^,^4^. Members of the chorion multigene family are grouped under α and β branches based on their evolutionarily conserved central domains, and further classified in three temporal groups according to the period of their expression. Thus, six types of chorion genes, early A, early B, middle A, middle B, late high-cysteine A (HcA) and late high-cysteine B (HcB), are clustered in the chorion locus of chromosome 2^5^. Moreover, most type A and type B genes are tightly paired and divergently transcribed under control of a common promoter region^6^,^7^,^8^.

The secreted chorion of *B. mori* is organized as a fibrous helicoidal array, yielding a lamellate structure which is constructed in four sequential morphogenetic modes^9^. During the early period of choriogenesis, a framework composed primarily of ErA and ErB chorion proteins forms a thin trabecular layer adjacent to the oocyte and a fibrous, helicoidal framework which encompasses the width of the eggshell. During the middle period, the framework undergoes expansion and densification by intercalation of newly synthesized class A and B chorion proteins. Finally, during the late period cysteine-rich HcA and HcB proteins penetrate and cross-link the entire chorion, and form a surface structure with denser helicoidal spacing^10^. After eggshell formation is completed, the follicular cells slough off.

Synthesis of chorion proteins begins in follicular cells (Supplementary Fig. 1B) just after vitellogenesis and continues to the end of choriogenesis. Recognizable classes of chorion proteins are synthesized sequentially with a high degree of developmental order^11^. The tissue-specific and temporally ordered expression make the chorion an excellent model system for research on gene regulation. For example, the common 5′-flanking region between pairs of coordinately expressed chorion genes belonging to A and B central domain families contains cis-regulatory elements responsible for chorion gene expression^12^ which share a chorion-specific hexanucleotide TCACGT essential for chorion gene expression^4^,^13^. Additionally, many transcription factors have been reported to be involved in controlling choriogenesis. For example, a GATA factor, isolated based on the presence of binding elements in the promoter sequences of late Hc chorion genes^14^,^15^, behaves as an early repressor of middle chorion genes; inhibition of its binding on promoter GATA-sites results in earlier expression of middle chorion genes^16^. Two types of C/EBP (a CCAAT/enhancer-binding protein) sites are also found in the chorion locus, an early type associated with early and early-middle chorion genes, and a late type for middle-late and late chorion genes, and a BmC/EBP factor has been shown to act both as an activator and a repressor during choriogenesis^17^. Nevertheless, these few regulatory components are too limited to account for the complex developmental patterns observed in choriogenesis.

Despite publication of many papers on chorion genes from the 1970s to the 1990s, many important features and the mechanisms underlying their regulation remained largely undetermined due to lack of knowledge of the complete landscape of the chorion locus and the relative unavailability of systems allowing a functional testing of specific hypotheses pertaining to the regulation of chorion gene expression. In 2008, an improved genome sequence of *B. mori* was published^18^; however, the chorion locus was largely interrupted by gaps due to the presence of repetitive sequences, resulting in an incomplete view of the region. In the present study, we pursued two approaches to determine the precise structure of the chorion locus. First, we performed an extensive EST analysis of a follicular cell cDNA library to identify chorion gene transcripts; second, using an available silkworm bacterial artificial chromosome (BAC) library, we produced a precise gene map of the chorion locus by constructing and sequencing a BAC contig covering it, and extending the analysis of its transcription. In addition, we conducted a direct analysis of eggshell protein components by liquid chromatograph-mass spectrometry (LC-MS). This is the first time that a complete chorion locus and its expression has been established on all of these levels, laying the foundation for a more comprehensive view of the developmental regulation of this complex multigene system.

## Results

### EST analysis of follicular cell and ovary cDNA libraries

To identify chorion gene transcripts, we analyzed ESTs of two newly constructed cDNA libraries, fcP8, derived from day 8 pupal follicular cells, and bmov, from day 4 pupal ovaries. From the fcP8 cDNA library, 643 ESTs were assigned to chorion gene transcripts, of which early, middle and late chorion transcripts comprised 27.5%, 35.8% and 36.7%, respectively. Amongst more than 20,000 ESTs from the bmov library, only 149 chorion transcripts were identified. These included 118 early and 31 middle but no late ESTs. Therefore, follicular cells from day 8 pupae were used to analyze gene expression throughout choriogenesis.

### Construction of a BAC contig and a complete sequence covering the chorion locus

**Figure 1:**
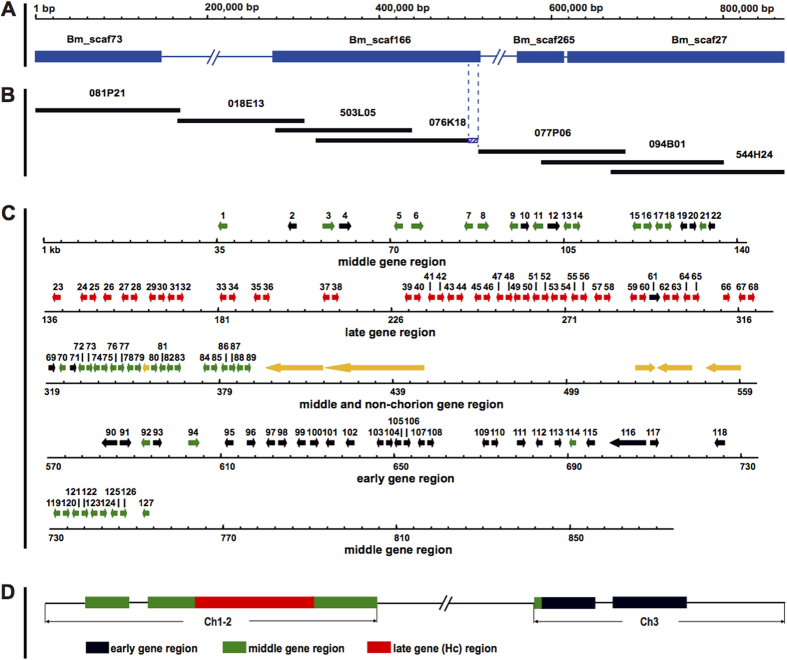
Illustration of the *Bombyx mori* chorion locus. (**A**) Diagram of the chorion locus in the *B. mori* genome assembly. Blue boxes and dotted lines represent scaffolds and gap regions edited from KAIKObase, respectively, 5′ is to the left. (**B**) BAC contigs that cover the chorion locus. Each black line represents a complete BAC region. Six BACs were sequenced except for 544H24, because its sequence was known. (**C**) Distribution of 127 chorion genes and 5 non-chorion genes in the complete chorion locus (871,711 base pairs). This figure presents a view with numbers and orientation of individual genes. Early, middle, late and non-chorion genes are highlighted in black, green, red and yellow, respectively. BmCho-mut located between genes 79 and 80 is also marked in yellow. Five sequence segments constitute the complete chorion locus. (**D**) Genomic overview of the previously published *B. mori* chorion locus^8^. Shown are early chorion genes and early region (black), middle chorion genes and middle region (green), late chorion genes and late region (red).

An incomplete genome assembly published in 2008 (Fig. 1A) showed that the chorion locus, with only 27 annotated chorion genes, was largely interrupted by three gaps due to the presence of repetitive sequences^18^. For this reason, we constructed new BAC contigs to cover the chorion locus, including the existing gaps. More than 200 BAC clones from early, middle and late chorion gene regions were screened with EST probes of representative chorion genes from the fcP8 cDNA library (Supplementary Table 1) by hybridization of a high-density replica (HDR) filter of the RPCI-96 silkworm BAC library. Among positive BAC clones, the highly positive BAC clones 077P06 and 094B01 were chosen for analysis of early chorion genes, 081P21 and 076K18 for middle chorion genes, and 018E13 for late chorion genes (Fig. 1B). We also selected clone 503L05, which had a strong positive signal and was known to cover a non-chorion domain of the locus based on its BAC end sequence, BES.503_L05 (acc. # DE379518), in KAIKObase (http://sgp.dna.affro.go.jp/KAIKObase/), and BAC 544H24, because we already knew that its full sequence was aligned with the 3′ part of the chorion locus and the neighboring region^18^. We performed contig construction for these BAC clones with the fingerprinting method described previously^19^. This resulted in two contigs: one composed of four BACs covering the 5′ half of the chorion locus, and a second made up of three BACs aligning with the 3′ half of the chorion locus (Fig. 1B). The 3′ end sequences of the 076K18 BAC contig and the 5′ end sequences of the 077P06 BAC contig (Fig. 1B) were located on the same scaffold, Bm_scaf166 (Fig. 1A). Thus, the previous gap between the two BAC contigs, which were connected on Bm_scaf166, covered the whole chorion locus (Fig. 1A,B).

Sequencing of these BACs (Fig. 1B) resulted in the deduction of the complete sequence of the chorion locus spanning 871,711 bp (DDBJ/GenBank/EMBL accession number AB999997) of chromosome 2. (nucleotides 1,748,531-3,958,793 in silkworm genome assembly v2 (http://sgp.dna.affrc.go.jp).

### Annotation of chorion genes

Using chorion ESTs and a gene prediction program^20^, 127 chorion genes were identified in the chorion locus (Fig. 1C; for specific details see Supplementary Table 2), which were named BmCho-1 to BmCho-127 in order from 5′ to 3′. The chorion genes were distributed as clusters in the genomic locus, where they were mainly paired according to gene family. These included 8 early A/B gene pairs, 15 middle A/B gene pairs, and 21 HcA/HcB gene pairs. Chorion gene coding sequences (CDS) were composed of a minor exon 1 (≈50 nt) and major exon 2 (300 nt–500 nt). The early A and early B introns were highly variable in length. In contrast, HcA and HcB genes harbored intron regions of approximately 80 nt and 480 nt, and middle A and B gene pairs BmCho-119/120, 121/122, 123/124 and 125/126 had intron lengths of 110 nt and 328 nt (+/−5 nt), while more variable intron lengths were present in other middle A and B gene pairs (82 nt–2436 nt). In terms of sequence similarities, the coding regions of the early chorion genes had low sequence identity (less than 50%). By contrast, the nucleotide identities for the coding regions of the minor exons averaged 81% for the middle A genes (range 41–100%) and 77% for the middle B genes (range 32–100%), and averaged 68% and 61% for middle A (range 36–97%) and B genes (range 30–96%) for the coding regions of the major exons. The coding regions of the HcA and HcB genes also had high levels of nucleotide sequence identity, average 82% (range 67–99%) and 73% (range 46–100%) for major exons of HcA and HcB, especially minor exon regions (average 95%; range 86–100%).

In addition, we examined the sequence conservation among Hc gene pairs and their surrounding flanking regions in more detail. We chose five gene pairs, BmCho-24/25, 27/28, 45/46, 47/48 and 49/50, since we could identify accurately the sequences of the corresponding gene region including the 3′ untranslated region (UTR) of HcB up to the 3′UTR of HcA and the 3′ flanking regions by comparison with full-length mRNA sequences for each gene. As flanking sequences, we used 300 bp immediately after the 3′UTRs. Sequence identity for the gene regions amounted to 86–96% among the five gene pairs, whereas for the 300 bp 3′ flanking sequences, identity fell to a range of 48–72%, which was very poor compared with the gene regions.

### Phylogenetic analysis

**Figure 2:**
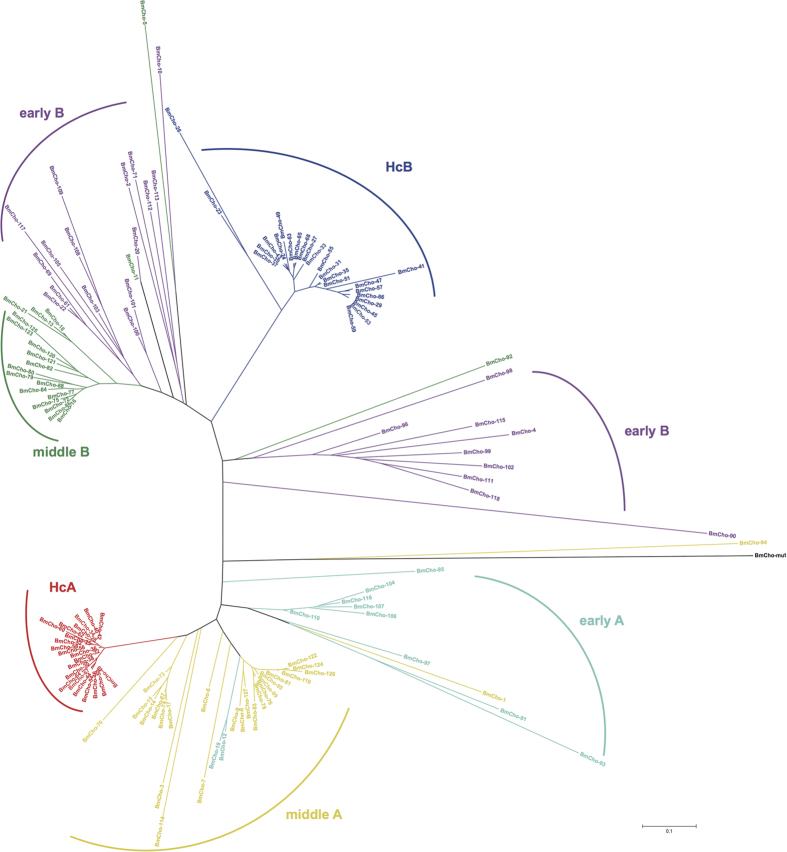
Evolutionary relationships of *Bombyx mori* chorion genes. The tree was constructed using the Neighbor-Joining and bootstrapping method in MEGA 6.06 (see Materials and methods). Different types of chorion genes are displayed by different colours.

**Figure 3:**
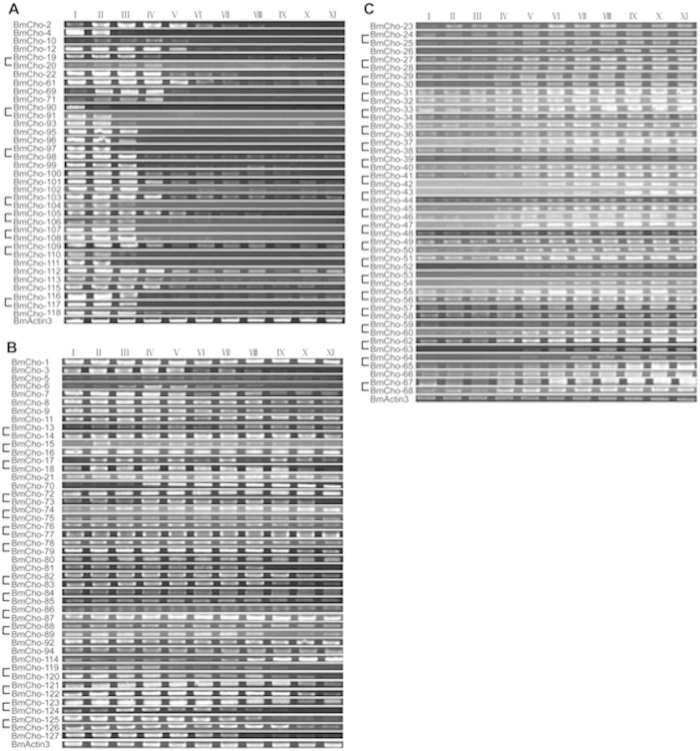
Expression patterns of early (**A**), middle (**B**) and late (**C**) chorion genes during choriogenesis. Gene pairs are marked by brackets. Roman numbers represent 11 stages of choriogenesis^3^.

Chorion genes are proposed to have originated from a common gene superfamily^21^. A phylogenetic analysis showed that the superfamily consisted of two distinct branches, A and B, each of them with differently diverged subfamilies (Fig. 2). Middle and late chorion genes were much more closely related to each other than to early chorion genes, which appeared to have undergone faster evolution than middle and late genes. In addition, early B genes were separated into two branches. One of two early B branches was more closely clustered with middle B genes, and 7 members of this branch (BmCho-22, BmCho-61, BmCho-100, BmCho-101, BmCho-103, BmCho-105 and BmCho-109) had an expression pattern distinct from the other early chorion gene group, with faint expression in middle and late stages of choriogenesis (Fig. 3A). BmCho-94 of middle A and BmCho-92 of middle B made a clade with the early genes in the phylogenetic tree.

### Insertion of a non-chorion region in the chorion locus

Our analysis also revealed that a 164 kb non-chorion region harboring five non-chorion genes between BmCho-89 and BmCho-90 existed in the chorion locus. The non-chorion genes encoded diacetylglycerol kinase beta (DKB), a partial DKB (may be a pseudogene), dihydrofolate reductase, ornithine decarboxylase antizyme and an unidentified protein product (Supplementary Table 2). To provide an estimate of the timing of insertion of the non-chorion region into the chorion locus, we investigated the conservation of synteny of orthologs of these non-chorion genes and several surrounding chorion genes in the genomes of *B. mori* and 4 other Lepidoptera, *Danaus plexippus, Heliconius melpomene, Manduca sexta* and *Plutella xylostella* (Supplementary Table 3). In *H. melpomene* and *M. sexta*, most of these non-chorion genes were located on the same scaffolds as the surrounding chorion genes; in *D. plexippus*, the dihydrofolate reductase and ornithine decarboxylase antizyme genes were located on a scaffold (DPSCF300489) containing 3 chorion genes, while other homologous chorion genes were located on a different scaffold (DPSCF300316). Altogether these data indicate that the non-chorion domains accompany chorion genes in two lepidopteran superfamilies (Bombycoidea and Papilionidae). For *P. xylostella* (Yponomeutoidea), a search in Diamondback moth Database^22^ revealed that chorion genes had a scattered arrangement in its genome. Nevertheless, 6 of 19 *P. xylostella* chorion genes (Px011664, Px011665, Px011667, Px011669, Px011671 and Px011674), were clustered on scaffold 446 adjacent to an ortholog of ornithine decarboxylase antizyme (Px011663). DKB (Px013844), DKB-like (Px013845) and dihydrofolate reductase (Px013846) were clustered on scaffold 59, but not associated with any chorion genes (Supplementary Table 3).

### Comparison of Hc gene regions in two silkworm strains

Comparison of the whole late chorion region between the present study and the previous report^8^ can provide information on differences in the strains used in the two projects. Initial detailed studies of chorion gene organization used strain 703, derived from a European strain^23^, which may have been separated at least 1,000 years ago^24^ from Dazao (equivalent to strain p50T used for the published genome sequence), a Chinese inbred strain used in the present study. In Dazao, we found a late chorion gene region of 180 kb containing 21 HcA-HcB gene pairs organized strictly head-to-head and separated by 330 nt (Supplementary Fig. 2A). This was interrupted only by a single early B chorion gene, BmCho-61, and three HcB genes. Two of the latter, BmCho-23 and BmCho-26, were pseudogenes, based on the presence of multiple stop codons in their coding regions (data not shown). Eighteen late chorion gene pairs (BmCho-24/25, 27/28–59/60), oriented HcB(−)/HcA(+), were located upstream of BmCho-61, followed by three late chorion gene pairs (BmCho-62/63, 64/65, 67/68) in reverse orientation as HcA(−)/HcB(+), and an unpaired HcB gene (BmCho-66) in reverse orientation from its neighbors. In contrast, in strain 703, 15 late HcA/HcB gene pairs were localized in a 140 kb region of Ch1-2 (Supplementary Fig. 2B). Here, 11 HcB(−)/HcA(+) gene pairs were located upstream of 6F6, encoding a B protein expressed in early choriogenesis, followed by four gene pairs in reverse orientation as HcA(−)/HcB(+), located at the end of the cluster. Additionally, three pseudogenes, including two HcBs and one HcA, were interspersed among the 15 gene pairs^25^,^26^,^27^,^28^. These differences preserved the general organization of the locus, including the presence of an early B gene that interrupted a long tandem array of HcA/HcB gene pairs, followed by a small group of HcA/HcB gene pairs in reverse orientation from the main cluster, but displayed some differences, such as the number and position of pseudogenes.

### Analysis of chorion transcription

We used a vitellogenesis-specific gene, vitelline membrane associated protein P30 (BmVMP30)^29^, as a staging marker for follicle transition from vitellogenesis to choriogenesis. RT-PCR confirmed that BmVMP30 indeed was only expressed in vitellogenesis but not in choriogenesis (Supplementary Fig. 3A; see Supplementary Table 4 for primer sequences), consistent with our staging. Nevertheless, we detected expression of some middle chorion genes in vitellogenesis using primers from exon 1 and exon 2 sequences (Supplementary Table 4). No unexpected bands were detected during that period except for BmCho-72, BmCho-77, BmCho-80 and BmCho-16 (Supplementary Fig. 3, panels B, C, D). The RT-PCR bands of these four genes obtained in vitellogenesis have been sequenced and found to be intron-containing splicing intermediates of middle and late chorion genes, not mature messenger RNAs (data not shown). Moreover, because of frequent stop codons, they could not be translated into functional chorion proteins during vitellogenesis. This conclusion was confirmed by the LC-MS study described below, which showed that expression of authentic chorion proteins is initiated just after vitellogenesis as previously reported^3^.

The expression profiles of the 127 chorion genes in choriogenic follicles by RT-PCR (Fig. 3; heatmaps in Supplementary Fig. 4) were determined using unique primers where possible (Supplementary Table 4), except for case where common primers for highly homologous sequences like some of the late chorion genes were used, due to the difficulty of designing unique primers. Early and late chorion genes were expressed exclusively during early and late choriogenesis, as expected (Fig. 3A,C). Surprisingly, transcripts of middle chorion genes were detected throughout choriogenesis (Fig. 3B). We further classified chorion genes by their central domain sequences (ErA, ErB, A, B, HcA, HcB) and expression patterns into 36 early, 46 middle, and 45 late chorion genes (Supplementary Table 2). Middle chorion genes included four early-middle genes, classified as such because of their expression in all stages except for late choriogenesis.

### Chorion transcript variants

**Figure 4:**
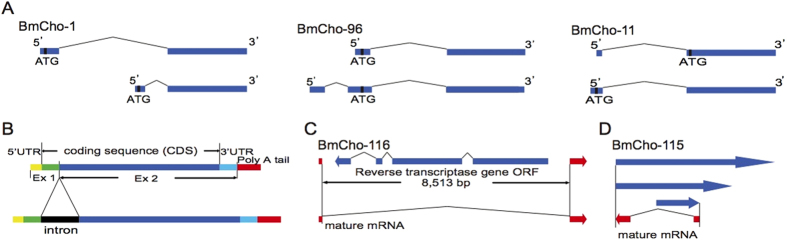
Structure of *Bombyx mori* chorion transcripts. **A**) Mature mRNAs of BmCho-1 and BmCho-96 in ovary/follicular cells (upper) and testis (lower). BmCho-11 illustrates the ovary/follicular cell-isoform (upper) and embryo-isoform (lower). Blue boxes, transcript sequences; thin lines, intron regions; first ATGs, vertical black lines. The precise sequence alignments are shown in Supplementary Fig. 5 (BmCho-1), Supplementary Fig. 6 (BmCho-96) and Supplementary Fig. 7 (BmCho-11). (**B**) Schematic diagrams of mature transcript (upper) and splicing intermediate (lower). Displayed are: coding sequence (CDS) (green and blue boxes), intron sequences (black box), 5′UTR (yellow box), 3′UTR (navy box) and poly A-tails (red box). Exon 1 and exon 2 are represented by Ex 1 and Ex 2, respectively. The mature transcript is comprised of 5′UTR, CDS, 3′UTR and poly-A tails; the splicing intermediate contains an intron sequence in addition to components of the mature transcript. (**C**) Diagram of BmCho-116 and reverse transcriptase gene. Blue box and arrow represent the exon of the reverse transcriptase gene and its transcriptional orientation; the thin line denotes the intron region. Red box and arrow represent the exon of BmCho-116. The mature BmCho-116 mRNA is represented by the lower diagram. (**D**) Illustration of BmCho-115 and its antisense RNAs. Red box and arrow represent the mature transcript of BmCho-115 and its transcriptional orientation, blue arrows indicate the antisense RNAs of BmCho-115 and their transcriptional orientation.

By conducting a search in a large EST database, SilkBase (http://silkbase.ab.a.u-tokyo.ac.jp/cgi-bin/), two chorion genes were found that had transcripts in testis. For BmCho-1 (Fig. 4A; Supplementary Fig. 5), follicular cell-derived and testis-derived transcripts shared the same second exon; however, the first exon was different in the two tissues. The first exon of the testis-derived transcript was located in an intron region of the ovary and follicular cell-derived transcripts, thus different proteins were conceptually encoded because of the different first exon sequences.

One of 13 independent clones encoding BmCho-96 found in SilkBase, clone ftes03K06, was also derived from testis, whereas the others were from ovary and follicular cells. The sequence of clone ftes03K06 was composed of three exons, while ovary and follicular cell transcripts had two exons, with a common last exon in both forms (Fig. 4A; Supplementary Fig. 6). The first exon of ftes03K06 was located in a 5′UTR of the ovarian transcript, but both types of transcripts shared the same ATG codon, resulting in the same CDS and putative proteins.

The gene for BmCho-11 (Fig. 4A; Supplementary Fig. 7) provided eight clones, three of which were derived from an embryonic cDNA library (fe8d; 200 hrs after fertilization), and the other five from a follicular cell library (fcP8). The follicular cell-derived transcripts contained the first methionine codon (ATG) near the beginning of exon 2; by contrast, exon 1 of the embryonic transcripts was longer and its first methionine codon was upstream of the follicular cell transcripts, although located in the same reading frame. Thus, the embryo-encoded protein was 18 amino acids longer than the one encoded by follicular cells.

Most of the aberrant high-cysteine transcripts, obtained from the follicular cell library (fcP8), were composed of a 5′UTR, CDS, intron, 3′UTR and poly A-tail. These transcripts (Fig. 4B) were probably splicing intermediates that could not produce proteins due to multiple stop codons, which arose from the presence of the intron.

A careful inspection of chorion gene intronic sequences uncovered an additional unexpected finding. The 8,513 bp intron of an early A chorion gene, BmCho-116, contained a complete Open Reading Frame (ORF) for reverse transcriptase (RT) encoded in the reverse orientation (Fig. 4C). We found two clones in the follicular cell cDNA library (fcP8; Supplementary Table 5), which were precursor mRNAs of this RT gene carrying poly A-tails. Two additional clones of BmCho-116 derived from the same cDNA library were splicing intermediates (Supplementary Table 5), whereas three clones derived from a pupal ovary cDNA library (bmov) were mature mRNAs (Supplementary Table 5).

A similar situation was observed for eight clones derived from another early chorion gene, BmCho-115. Among six clones derived from the follicular cell library (fcP8; Supplementary Table 5), three encoded antisense RNAs containing intron sequences (Fig. 4D), while three encoded mature chorion sequences transcribed from the complementary DNA strand (Supplementary Table 5). In contrast, two clones obtained from the pupal library encoded mature early cDNA sequences (Supplementary Table 5).

### Proteome analysis of the eggshell

Using LC-MS, 260 proteins were identified in the intact eggshell, of which 99 were authentic chorion proteins (Supplementary Table 6). The protein products of the remaining 28 chorion genes were not detected, possibly due to low abundance, or encoding by pseudogenes with multiple stop codons which did not produce identifiable peptides. Twenty-three ovary-specific non-chorion proteins were also identified in the eggshell; most of the corresponding genes were clustered on chromosomes 2 and 15^30^. A chorion-like protein was also detected by LC-MS (Supplementary Table 6), whose gene (Gene007414) was located on chromosome 12. This gene had the same expression pattern as early chorion genes during choriogenesis (Supplementary Fig. 3A; primers are shown in Supplementary Table 4), indicating that the protein was likely involved in forming the early framework of the eggshell.

By comparison between dissected portions of the eggshell with and without the micropyle, we looked for evidence of localized expression of chorion proteins. We identified seven chorion proteins that were present only in the micropyle portion. Among these, six were high-cysteine chorion proteins (BmCho-24, 25, 32, 34, 36, and 58), and one was early (BmCho-118).

### Analysis of cis-elements in chorion gene promoters

Using a high-quality transcription factor binding profile database (http://jaspar.genereg.net/), we analyzed two key cis-elements, for GATA and C/EBP factor binding, in the bidirectional promoter regions of chorion gene pairs^31^,^32^ and within 2 kb upstream of all the unpaired and functional chorion genes. This analysis revealed the existence of these two cis-elements, in agreement with previous reports^16^.

The C/EBP element, 5′-TKNNGY/AAAK/C-3′ (K = T or G, Y = T or C)^4^,^33^, existed in all common promoter regions. Similarly, typical GATA sites of 5′-WGATAR-3′ (W = A or T, R = A or G)^14^,^34^, could be found in all common promoter regions except those of 4 middle gene pairs: BmCho-119/120, 121/122, 123/124, 125/126 (Supplementary Fig. 8). In addition, the common promoter region between the two TATA boxes of HcA/HcB gene pairs was highly conserved, with over 90% identity. Fifteen middle A/B gene pairs were assigned to three groups depending on the sequence similarities of the common promoter regions between the TATA boxes, which showed over 80% identity (Supplementary Fig. 9). However, sequence identity of the common promoter region was low (less than 50%) among the early A/B gene pairs.

In two unpaired pseudogenes, BmCho-23 and BmCho-26, which were homologous to HcB chorion genes but bore multiple single nucleotide substitutions introducing termination codons in the coding regions, putative C/EBP and GATA sites were also identified. These were present in the 2 kb region upstream of the translational start sites of the two putative pseudogenes.

## Discussion

We have successfully obtained a complete continuous sequence of the chorion locus. To accomplish this, we constructed a BAC contig of 871,711 bp, which harbors 127 tandemly arrayed chorion genes organized in two segments flanking a 164 kb region with 5 non-chorion genes. The overall organization of the chorion locus reported here, including the composition of the gene clusters and the intercalation of a segment lacking chorion genes, is similar in broad outline to earlier genetic and physical maps. The relatively long non-chorion segment and differences in the silkworm strains used by various projects explain the discontinuity of the earlier genetic^35^ and physical^8^ maps and minor differences in chorion gene composition. The genetic map, which was constructed at low resolution using protein polymorphisms as markers, has two chorion gene clusters, Ch1-2, containing middle and late genes, and Ch3, containing mainly early genes, separated by 4.0 centiMorgans (cM), which is likely the non-chorion segment. The physical map (Fig. 1D)^8^, which was reported in 4 discontinuous segments totalling nearly 500 kb, was based on chromosome walking using relatively short lambda and cosmid clones^8^,^25^ screened with plasmids carrying chorion sequences, and therefore, unable to link together some chorion genes, or span segments lacking them. The failure to find linkage of many chorion genes in *P. xylostella* may be the result of a fragmented genome assembly rather than fundamental differences in gene organization. Nevertheless, the conserved linkage of at least some genes found in the non-chorion region to chorion homologs in 4 ditrysian families indicates that this segment was inserted into the chorion locus before separation of the Yponomeutoidea, which have an estimated divergence time of 40 MY from *Bombyx* and *Danaus*, a representative butterfly^36^.

Comparison of the Hc gene region between the two silkworm strains, Dazao, used here, and 703, used previously, revealed retention of an inversion and insertion of an early middle gene, an increased number of Hc gene pairs in Dazao, and a high degree of conservation of coding and flanking non-coding regions. It is suggested that these differences are the result of gene duplication events^37^ with subsequent accumulation of mutations and gene conversion^8^. Such evolutionary changes are commonly observed in tandemly arrayed multigene families and likely to have occurred during the estimated 1,000-year separation of the two silkworm strains^24^.

Previous reports had suggested that Hc genes have been subjected to gene conversion events at high rates^7^,^26^, a notion consistent with the present phylogenetic analysis. However, the sequences of two pseudogenes, BmCho-23 and BmCho-26 (Fig. 2 and Supplementary Table 2), suggested a faster evolution relative to other functional Hc genes. Moreover, a newly characterized gene, BmCho-mut (Fig. 1C), apparently encoded originally a middle A chorion protein; however, accumulation of mutations causing frame-shifts resulted in the encoding of a non-chorion protein. The phylogenetic analysis suggested that BmCho-mut has also undergone fast evolution. In addition, pseudogene transcripts have been identified in follicle cells suggesting that their 5′-flanking sequences were still functionally active^38^. These observations further support the hypothesis that chorion pseudogenes are not simply dead-ends, but can contribute directly to the evolution of the locus by serving as templates for gene conversion^39^.

Phylogenetic analysis also suggests that early chorion genes underwent faster evolution than middle and late genes, especially early B genes. Figure 1C provides information that can take account of concerted evolution in the evolutionary history of this multigene family. For example, middle A gene BmCho-94 and middle B gene BmCho-92 are grouped with early genes in the phylogenetic tree. Since they are surrounded by early genes in the genome, this suggests that gene conversions may have generated their correspondence with early gene sequences. Early A genes BmCho-12 and BmCho-19 are grouped in class A in the phylogenetic tree, which would be just a reverse event.

The extensive structural analysis of the chorion locus has yielded evidence for a variety of potential mechanisms regulating chorion gene expression, including antisense RNA^40^, splicing variants^15^, alternatively polyadenylated isoforms and distinct promoters for different tissues. Thus, the middle genes BmCho-1 and BmCho-11, and early gene BmCho-96 (Fig. 4A) are regulated by differential promoter usage and alternative splicing in follicular cells and testes (BmCho-1 and BmCho-96) or embryos (BmCho-11). Two examples of antisense RNAs involving early genes, BmCho-116 and BmCho-115, are also reported here. For BmCho-116, only mature mRNAs were found in the day 4 pupal follicular cells (bmov) library, whereas intron-containing antisense RNA was identified in the day 8 pupal library (fcP8). These observations suggest that the splicing and maturation of BmCho-116 precursor mRNAs proceed normally when early chorion genes are mainly expressed (bmov library), whereas splicing may be blocked by antisense RNA transcribed from the RT gene located in the BmCho-116 intron later in choriogenesis (fcP8 library). For BmCho-115, again, antisense RNAs were also identified only in the library that contained a complete set of choriogenic follicles (fcP8). This finding further supports the idea that the intron sequences of these two chorion genes may be involved in the regulation of their expression. The existence of splicing intermediates in most Hc genes suggests that intronic sequences within splicing intermediates or splicing intermediates themselves may play a role in the regulation of expression of Hc chorion genes. In addition, alternative polyadenylation isoforms were identified in the early chorion gene transcripts of BmCho-98, BmCho-99 and BmCho-100, which may regulate expression of chorion genes in an as yet undefined way.

In conclusion, we have established an updated and expanded view of the chorion locus, which includes the organization, annotation and structures of 127 chorion and 5 non-chorion genes and their expression profiles, and provided a comprehensive set of proteomic data for the eggshell. The precise landscape of the chorion locus together with the detailed characterization of the distinct transcriptional profiles of the different chorion genes during choriogenesis should now provide a new impetus for re-focussing attention on silkmoth chorion genes as one of the best models for understanding the spatial and temporal control of expression of eukaryotic multigene families. Last but not least, the information provided here should accelerate the positional cloning of several known chorion Grey (Gr) egg mutations^23^,^41^ and open up new avenues for breed improvement.

## Materials and Methods

### Insects and collection of samples

*B. mori* (Dazao) larvae were reared on fresh mulberry leaves or artificial diet in a dedicated silkworm rearing room. For collecting follicles, we used two characteristics to mark the first and last choriogenic follicle, shiny vitelline membrane and sloughing off of follicular cells, respectively (Supplementary Fig. 1A). For choriogenesis staging, we partitioned the choriogenic period into 11 stages as described previously^3^ (Supplementary Fig. 1A). Choriogenic follicles at the same stage from a single day 8 pupa were pooled and directly collected in Trizol (TaKaRa) for mRNA extraction according to the manufacturer’s instructions. Procedures were carried out at room temperature (approximately 22–24 °C) and generally completed within 2 hr after initial pupal dissection. We believe our measurements are comparable to results of earlier studies performed under similar conditions in which the developmental timing of eggs along an ovariole was estimated to be approximately 8.6 hr for each stage^3^. Eggshells for protein extraction and LC-MS analysis were dissected from virgin adults. A sharp scalpel was used to collect eggshell samples containing micropyles and without micropyles. Eggshells were washed using distilled water until the water was pellucid to avoid contamination by oocyte proteins in a 20 °C constant temperature room. Supplementary Fig. 1 shows the relationship between follicular epithelial cells and chorion.

### Full-length cDNA and EST library construction

Full-length and standard cDNA libraries, bmov and fcP8, were constructed from day 4 pupal ovary, which contain immature follicles that have not initiated choriogenesis, and day 8 pupal follicular cells, which include all stages of choriogenesis, as described previously^30^. All ESTs derived from the bmov and fcP8 cDNA libraries were deposited in DDBJ/GenBank/EMBL databases under accession numbers FY000001-FY021573 for bmov and BY918786-BY920388, BY927072-BY928825 for fcP8.

### BAC screening

The silkworm BAC library (RPCI-96) used in this paper was obtained from BACPAC Resources Center, Children’s Hospital, Oakland Research Institute and previously described^19^,^42^. BAC clones derived from the chorion locus were screened by hybridization of BAC HDR filters arrayed in duplicate with RPCI-96 BAC clones (BACPAC Resources Center [ http://bacpac.chori.org/]) with chorion gene probes. Labeling, hybridization and detection were as described previously^19^.

### Genomic sequencing

BAC DNA was extracted using a Large-Construct Kit (QIAGEN) in accordance with the manufacturer’s instructions. Two kilobase and five kilobase shotgun libraries for each BAC were constructed using a pUC118 vector. For each library, 590 clones were picked for bidirectional sequencing performed with an ABI3730 DNA Analyzer (Applied Biosystems). After trimming vector sequences, all paired-end reads were assembled with the programs Phrap 1.08081222^43^ and Consed 16.0^44^.

### RT-PCR

Total RNA was subjected to reverse transcription using a PrimeScript™ RT Master Mix (Perfect Real Time) (TaKaRa) in 50 μl reaction volume (2500 ng total RNA) and then diluted 5-fold. One μl cDNA was used in 10 μl PCR reaction volume. PCR was carried out with the following program: 94 °C for 2 min followed by 30 cycles of 94 °C for 10 sec, 50 °C for 15 sec, and 72 °C for 30 sec with rTaq DNA polymerase (TaKaRa) using chorion-specific primers listed in Supplementary Table 4. RT-PCR of each chorion gene was repeated at least three times in two independent samples. Representative samples are presented in Fig. 2. BmActin3 was used as a standard for each set of PCR reactions and for gel loading.

### Phylogenetic analysis

ClustalX 1.83 was used to generate alignments of protein sequences^45^. Chorion protein sequences were subjected to phylogenetic analysis by neighbor-joining and bootstrapping methods with 1000 replicates using MEGA6.06^46^.

### LC-MS

Chorion samples with and without micropyle were extracted with 8 M urea/100 mM DTT buffer. Protein concentrations were determined using the Bradford method^47^. Equal amounts of two corresponding samples were digested using the filter aided sample preparation method^48^, and 5 μg peptides of each sample were loaded, then peptides were separated successively on Thermo scientific EASY columns (2 cm*100 μm 5 μm-C18 and 75 μm*100 mm 3 μm-C18). MS/MS analysis was performed on a Q Exactive mass spectrometer (Thermo Finnigan). To annotate chorion proteins, raw files were analyzed using Mascot 2.2 and searched against the *B. mori* comprehensive gene set (http://sgp.dna.affrc.go.jp/ComprehensiveGeneSet/).

## Additional Information

**How to cite this article**: Chen, Z. *et al.* A comprehensive analysis of the chorion locus in silkmoth. *Sci. Rep.* **5**, 16424; doi: 10.1038/srep16424 (2015).

## Supplementary Material

Supplementary Information
