# Supplementary material for: A comprehensive analysis of the chorion locus in silkmoth
Source: Sci Rep. 2015 Nov 10;5:16424. doi: 10.1038/srep16424 (PMC4639761; doi:10.1038/srep16424)
Supplement: Supplementary Information [file srep16424-s1.pdf]

A comprehensive analysis of the chorion locus in silkmoth

Zhiwei Chen[1], Junko Nohata[2], Huizhen Guo[1], Shenglong Li[1], Jianqiu Liu[1], Youbing Guo[1], Kimiko Yamamoto[3], Keiko Kadono-Okuda[3], Chun Liu[1], Kallare P. Arunkumar[4], Javaregowda Nagaraju[4†], Yan Zhang[1], Shiping Liu[1], Vassiliki Labropoulou[5], Luc Swevers[5], Panagiota Tsitoura[5], Kostas Iatrou[5]*, Karumathil P. Gopinathan[6], Marian R. Goldsmith[7]*, Qingyou Xia[1] & Kazuei Mita[1]*

[1]State Key Laboratory of Silkworm Genome Biology, Chongqing 400716, China. [2]Kirin Brewery Co. Ltd, Toride, Japan. [3]National Institute of Agrobiological Sciences, Tsukuba 305-8634, Japan. [4]Centre for DNA Fingerprinting and Diagnostics, Hyderabad 500001, India. [5]Insect Molecular Genetics and Biotechnology, Institute of Biosciences & Applications, National Centre for Scientific Research "Demokritos", Athens 15310, Greece. [6]Indian Institute of Science, Bangalore, India. [7]University of Rhode Island, Kingston 02881, USA.

† deceased

To whom correspondence should be addressed: State Key Laboratory of Silkworm Genome Biology, Chongqing 400716, China. E-mail: mitakazuei@gmail.com. Tel: +86-18983794244. Fax: +86-023-68250892; Department of Biological Sciences, University of Rhode Island, Kingston, Rhode Island 02881, USA. E-mail: mki101@uri.edu. Tel: 1-401-874-2637. Fax: 1-401-874-2065; and Insect Molecular Genetics and Biotechnology, Institute of Biosciences & Applications, National Centre for Scientific Research "Demokritos", Athens 15310, Greece. E-mail: iatrou@bio.demokritos.gr. Tel: +30-210-650-3562; Fax: +30-210-651-1767

Supplementary information, Figure S1

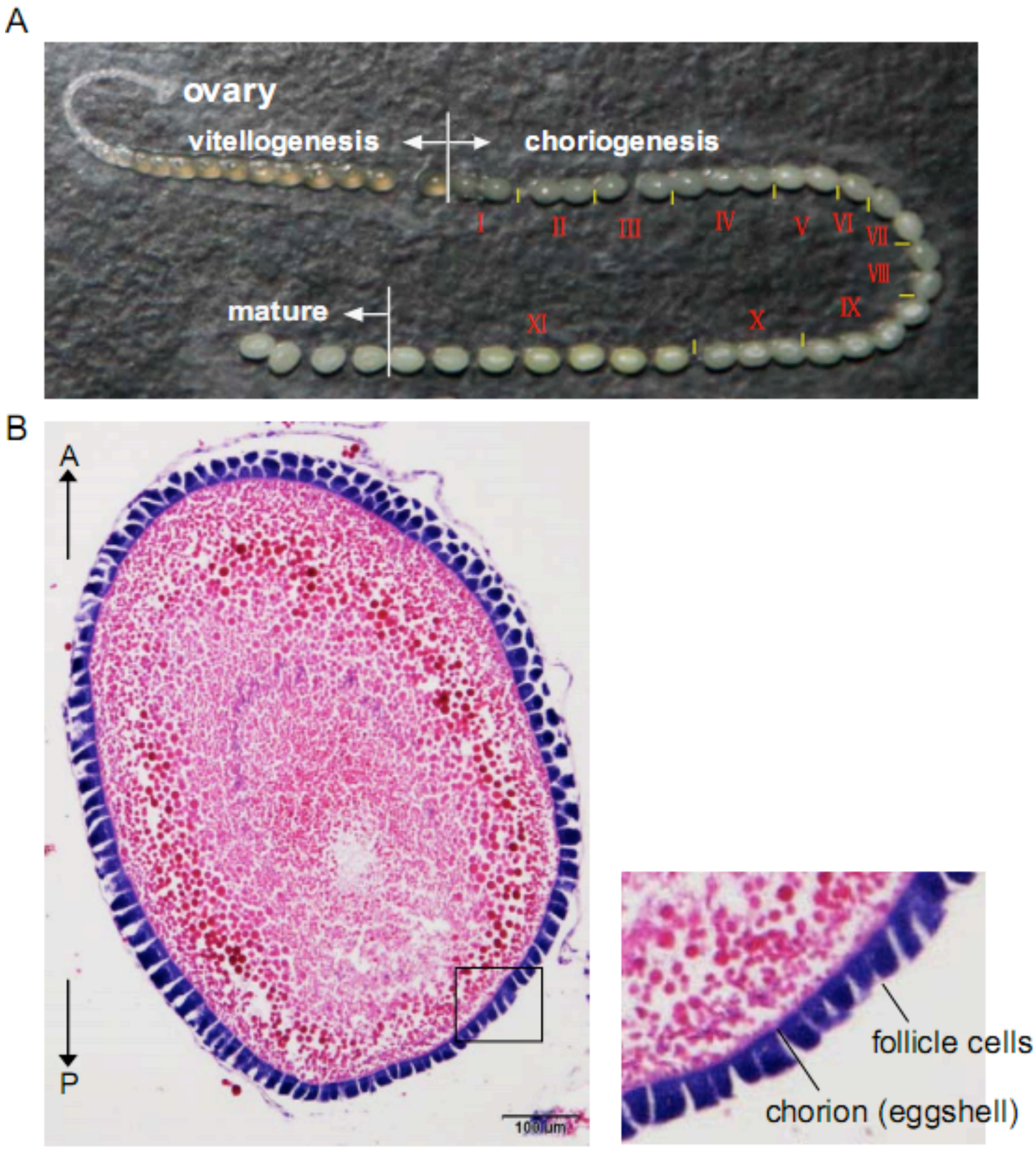

Supplementary Figure 1. The ovary, follicle cells and chorion (eggshell) in silkworm. A. The developing oocytes (eggs) from day 8 papae. The ovary is located at the beginning of ovariole. B. Hematoxylin Eosin (HE) staining for oocyte which comes from choriogenesis stage. The letter A indicates anterior pole of egg, and the P indicates posterior pole of egg. The passageway (micropyle) for sperm is formed at the anterior cap, traversing the chorion to permit fertilization. The follicle itself

consists of a single layer of polyploid epithelial cells (follicle cells) which surrounds the chorion of oocyte.

Supplementary information, Figure S2

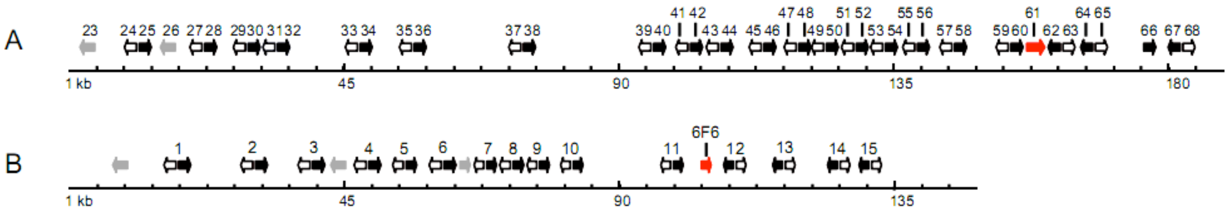

Supplementary Figure 2. Comparison of Hc gene pairs between strain 703 and Dazao. A. Distribution of Hc gene pairs with Gene ID in Dazao. B. Distribution of Hc gene pairs with numbers in strain 703. The gray arrows indicate psedogenes.

Supplementary information, Figure S3

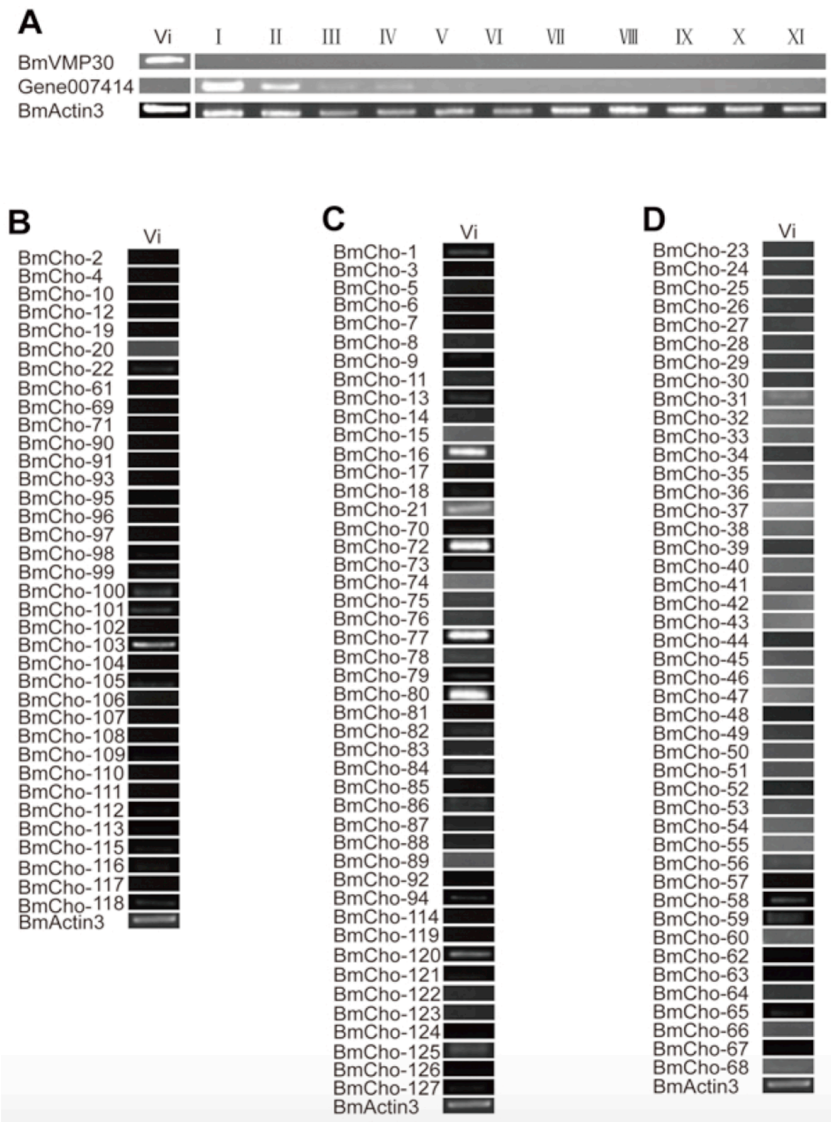

Supplementary Figure 3. Expression analysis of BmVMP30, Gene007414 and chorion genes. A. Expression pattern of BmVMP30 and Gene007414 in vitellogenesis and choriogenesis. B, C, D. Expression of early (B), middle (C) and late (D) chorion genes in vitellogenesis. BmActin 3 was used as a standard for PCR reactions and gel loading.

Supplementary information, Figure S4

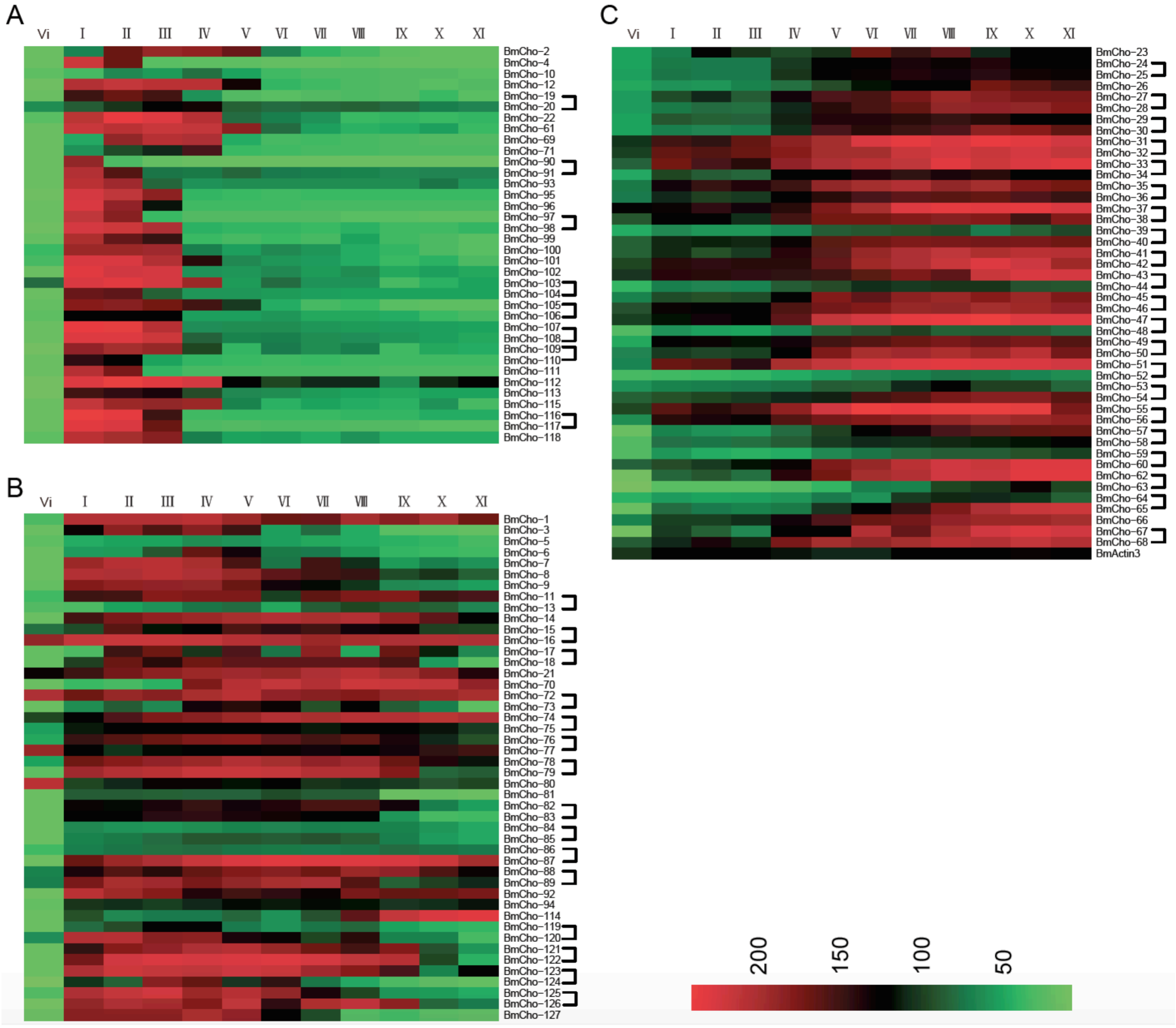

Supplementary Figure 4. Heat maps for expression pattern of early (A), middle (B) and late (C) chorion genes during vitellogenesis and choriogenesis. Vitellogenesis is denoted as Vi; Roman numbers represent 11 stages of choriogenesis. Gene pairs are marked by brackets.

Supplementary information, Figure S5

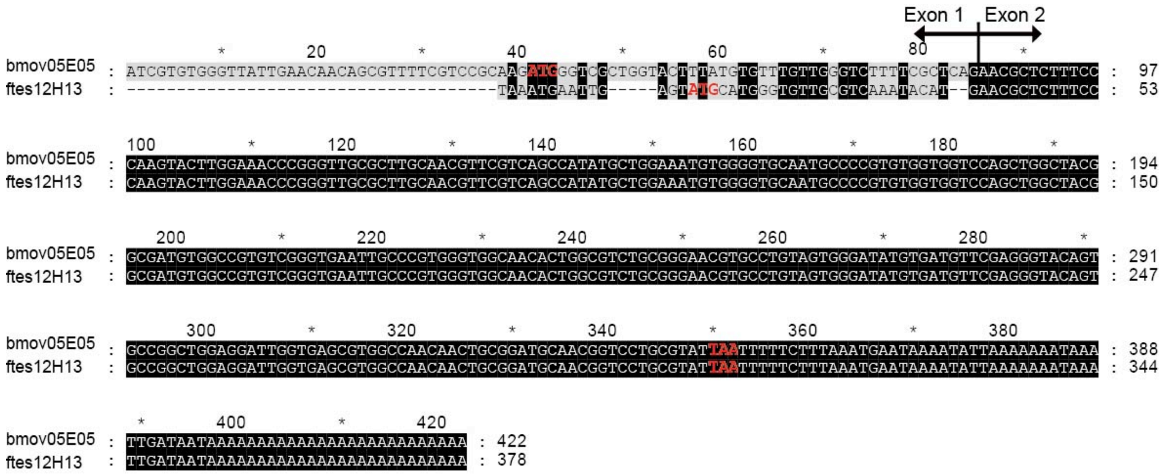

Supplementary Figure 5. Sequence alignment of ovary- and follicular cell-derived BmCho-1 (bmov05E05) and testis-derived BmCho-1' (ftes12H23). The first methionine codon ATG and stop codon TAA are highlighted in bold and red. Exon 1 and exon 2 are demarcated by vertical black lines.

Supplementary information, Figure S6

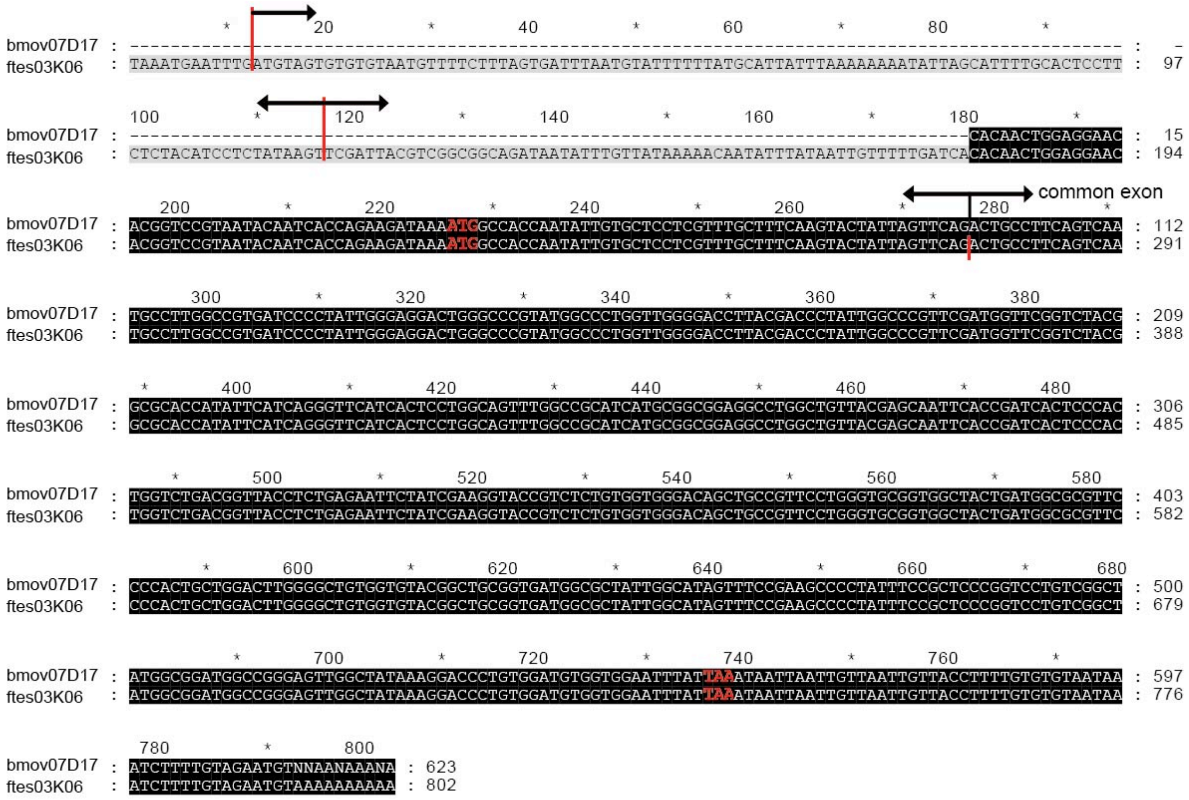

Supplementary Figure 6. Sequence alignment of ovary- and follicular cell-derived BmCho-96 (bmov07D17) and testis-derived BmCho-96' (ftes03k06). The first methionine codon ATG and stop codon TAA are highlighted in bold and red. Exons are demarcated by vertical black lines for bmov07D17, and vertical red lines for ftes03K06.

Supplementary information, Figure S7

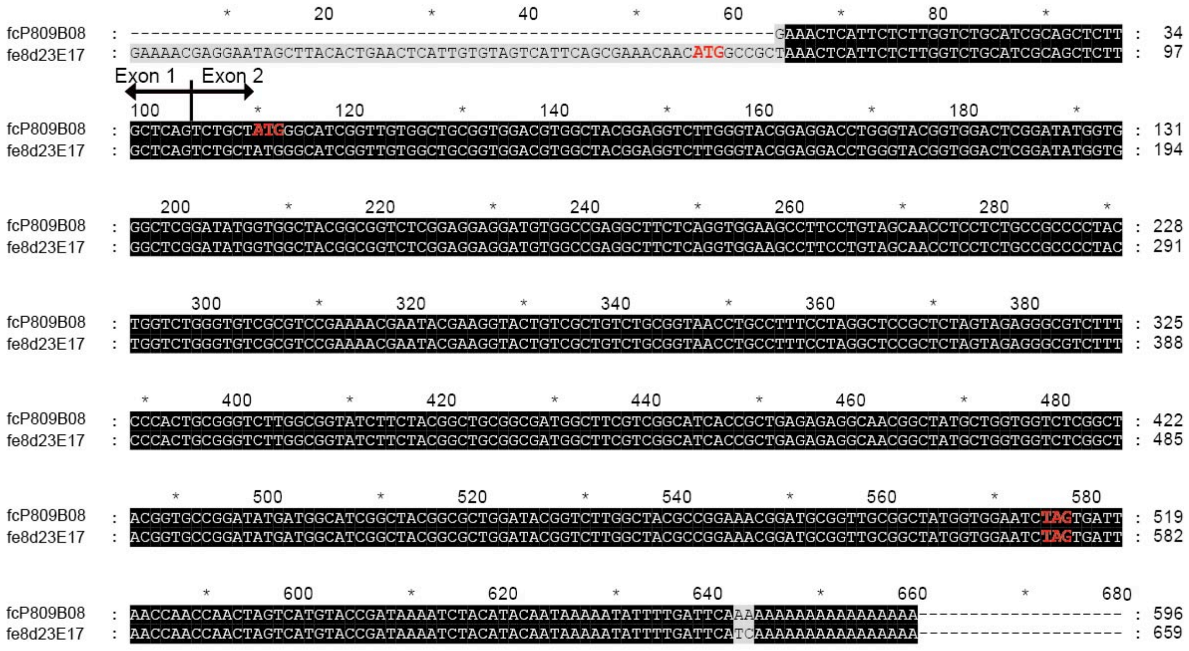

Supplementary Figure 7. Sequence alignment of ovary- and follicular cell-derived BmCho-11 (fcP809B8) and embryo-derived BmCho-11' (fe8d23E17). The first methionine codon ATG and stop codon TAG are highlighted in bold and red. Exon 1 and exon 2 are demarcated by vertical black lines.

Supplementary information, Figure S8

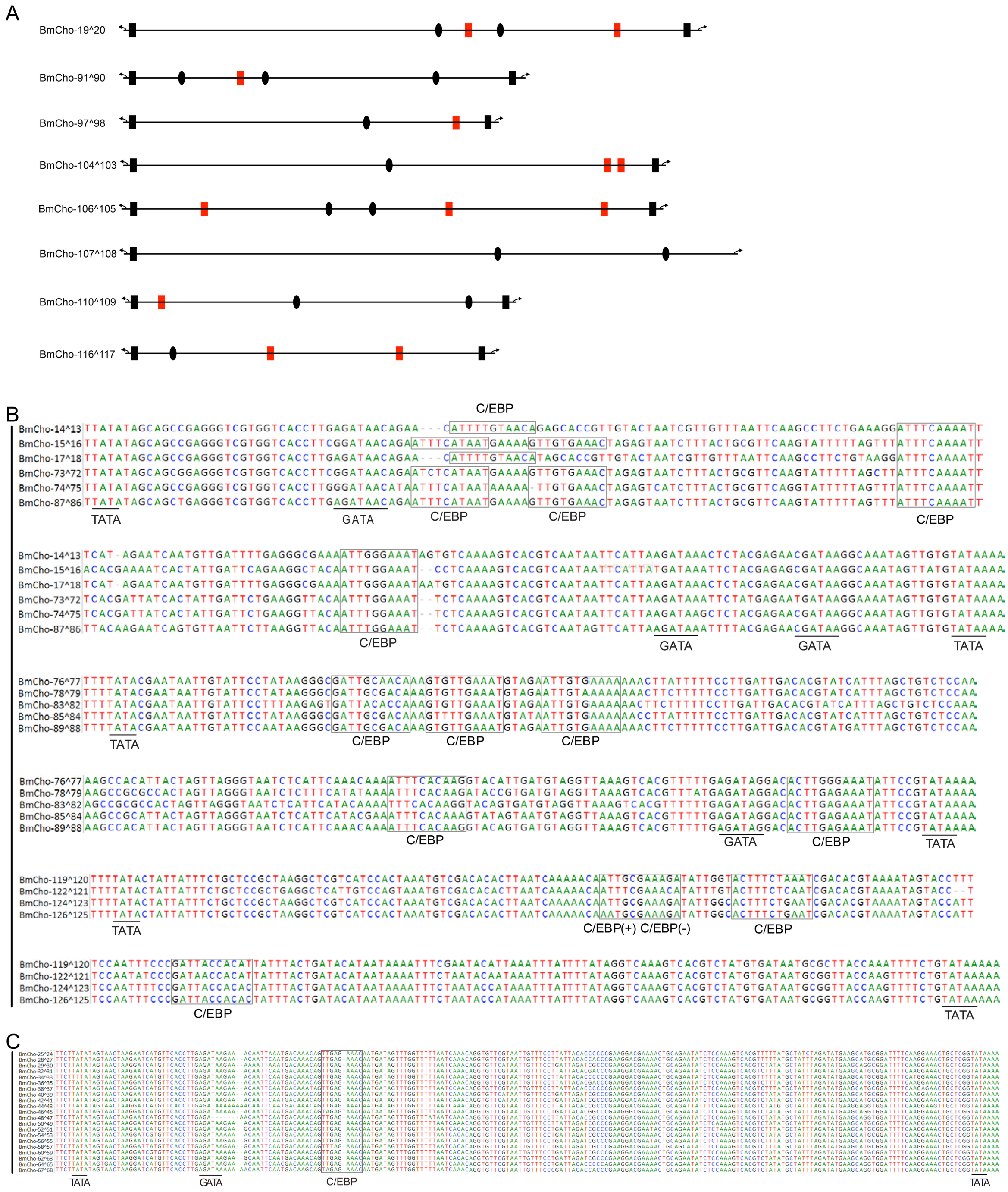

Supplementary Figure 8. Distribution of two key cis-elements (GATA and C/EBP) in common promoter regions of early (A), middle (B) and late gene pairs (C). Red box indicates

the element of GATA; black oval indicates the element of C/EBP; black box indicates the TATA box.

Supplementary information, Figure S9

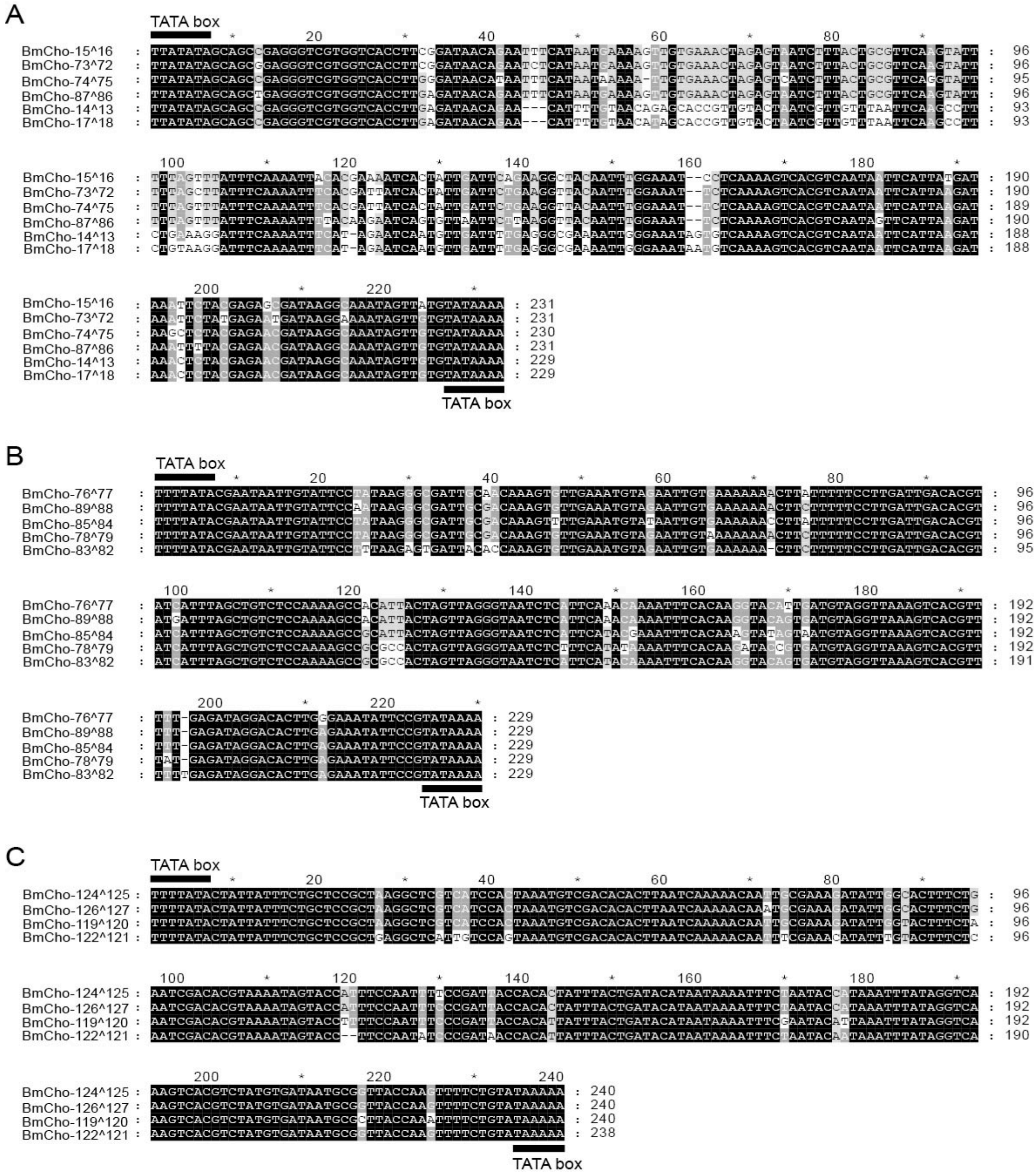

Supplementary Figure 9. Alignment of common promoter sequences between TATA boxes of middle chorion gene pairs. The common promoter sequences between TATA boxes of middle chorion gene pairs were divided into three groups (A, B and C) according to sequence homology.

Supplementary information, Table S1. ESTs used as probes for screening BAC clones.

Type of chorion	Gene ID	cDNA clone	accession #
early A	BmCho-110	fcP809F08	BY919409
early B	BmCho-109	fcP807G07	BY927624
Middle A	BmCho-9	fcP812C07	BY919605
Middle B	BmCho-11	fcP809B08	BY919370
Middle A	BmCho-76	fcP814C12	BY919762
Middle B	BmCho-82	fcP818B03	BY920045
Late HcA	BmCho-28	fcP802B12	BY927183
Late HcB	BmCho-33	fcP815F06	BY919864
Late HcA	BmCho-64	fcP816D07	BY919923
Late HcB	BmCho-65	fcP806G06	BY919206

Supplementary information, Table S2. Supplementary Table S2. Summary of Bombyx mori chorion and non-chorion gene information for the complete chorion locus.

Table S2 was provided as single pdf format.

Supplementary information, Table S3. Synteny of non-chorion genes and neighboring chorion genes in the chorion locus in *B. mori*, *Danaus plexippus*, *Heliconius melpomene* and *Manduca sexta*.

Bombyx mori					Homologous gene in Danaus plexippus		Homologous gene in Heliconius melpomene		Homologous gene in Manduca sexta		Homologous gene in Pletella xylostella	
chr.	chr start	chr end	Annotation	Type of chorion	Gene ID (Dp)	Scaffold ID	Gene ID (Hm)	Scaffold ID	Gene ID (Ms)	Scaffold ID	Gene ID (Px)	Scaffold ID
chr2	1789026	1790731	BmCho-1	middle A	DPOGS206761	DPSCF300316: 110139-110579			Msex002646-RA	scaffold00032: 1611237-1612178	no hit	
chr2	1816857	1819415	BmCho-4	early B	DPOGS206741	DPSCF300316: 105328-106349			Msex004538-RA	scaffold00064: 36696-46382	no hit	
chr2	2531413	2548398	Diacetylglycerol kinase beta		DPOGS201826	DPSCF300482: 18672-28307	HMEL011198	HE671164:399805-409344	Msex004556-RA	scaffold00064: 150764-226783	Px013844	scaffold_59:623742-675032
chr2	2549161	2637964	Diacetylglycerol kinase 1		DPOGS201825	DPSCF300482: 29341-39097			Msex004556-RA	scaffold00064: 150764-226783	Px013845	scaffold_59:688513-693487
unmapped	Bm_scaf199: 8451-12057		dihydrofolate reductase		DPOGS212793	DPSCF300489: 12250-16876	HMEL011200	HE671164:467099-471590	Msex004555-RA	scaffold00064:138119-144237	Px013846	scaffold_59:748748-753433
unmapped	Bm_scaf199: 15839-34570		Ornithine decarboxylase antizyme		DPOGS212792	DPSCF300489:	HMEL011201	HE671164:472348-477238	Msex004554-RA	scaffold00064:115608-130294	Px011663	scaffold_446:144443-165722
chr2	3167652	3172136	BmCho-90	early B	no hit				Msex004541-RA	scaffold00064: 61641-62838	no hit	
									(Msex002647-RA)		no hit	
chr2	3172326	3174950	BmCho-91	early A	no hit				no hit		no hit	
chr2	3176981	3177553	BmCho-92	middle B	DPOGS205110	DPSCF300172: 53024-53591	HMEL013746	HE671386:147726-148506	Msex004548-RA	scaffold00064: 84550-89237	no hit	
chr2	3178845	3180714	BmCho-93	early A	DPOGS206743	DPSCF300316: 944-98105	HMEL011169	HE671164:146050-146557	Msex004543-RA	scaffold00064: 1594555-1602943	no hit	
					DPOGS212790	DPSCF300489: 56351-58939	HMEL011206	HE671164:524426-525232	(Msex002648-RA)	(scaffold00032: 1615556-1616711)	no hit	
chr2	3182811	3197984	BmCho-95	early A	DPOGS206743	DPSCF300316: 944-98105	HMEL011169	HE671164:146050-146557	Msex004543-RA	scaffold00064: 1594555-1602943	no hit	
					DPOGS212790	DPSCF300489: 56351-58939	HMEL011206	HE671164:524426-525232	(Msex002648-RA)	(scaffold00032: 1615556-1616711)	no hit	
chr2	3187669	3189743	BmCho-94	middle A	DPOGS212797	DPSCF300489: 51236-53388	HMEL011202	HE671164:490645-491524	Msex004550-RA	scaffold00064: 91142-92053	no hit	

Supplementary information, Table S4. Supplementary Table S3 Chorion gene primers used for RT-PCR.

Table S4 was provide as single pdf format.

Supplementary information, Table S5. Variant clones obtained for BmCho-115 and BmCho-116 from follicular (fcP8) and ovarian (bmov) cDNA libraries.

**Chorion gene**	**Variant type**	**clone ID**	**Accession number**
BmCho-116	RT precursor with polyA tail	fcP803F10	acc. # BY918994
		fcP821B03	acc. # BY920258
BmCho-116	Splicing intermediates	fcP801C02	acc. # BY918804
		fcP809D09	acc. # BY919389
BmCho-116	Mature mRNA	bmov01E07	acc. # BY000095
		bmov02J22	acc. # BY000573
		bmov14E19	acc. # BY994730
BmCho-115	Antisense RNA with intron	fcP816C08	acc. # BY928301
		fcP816D04	acc. # BY928309
		fcP819C05	acc. # BY928544
BmCho-115	Mature chorion mRNA in bmov	bmov03I18	acc. # FY000893
		bmov11H02	acc. # FY003707
BmCho-115	Mature chorion mRNA in fcP8	fcP804A09	acc. # BY927335
		fcP820A01	acc. # BY928595
		fcP821G09	acc. # BY928738

Supplementary information, Table S6. Eggshell proteins of *B. mori* detected by LC-MS.

Table S6 was provided as single pdf format.
